# Sebaceous Hyperplasia of the Face: A Case Report

**DOI:** 10.7759/cureus.71196

**Published:** 2024-10-10

**Authors:** Deeksheetha Prabhu Venkatesh, Karthikeyan Ramalingam, Pratibha Ramani, Rajprakash Bhaskaran

**Affiliations:** 1 Oral Pathology and Microbiology, Saveetha Dental College and Hospitals, Saveetha Institute of Medical and Technical Sciences, Saveetha University, Chennai, IND; 2 Oral and Maxillofacial Surgery, Saveetha Dental College and Hospitals, Saveetha Institute of Medical and Technical Sciences, Saveetha University, Chennai, IND

**Keywords:** dermatology, face, forehead, hyperplasia, pathology, sebaceous, sebaceous glands, sebaceous hyperplasia, skin, skin lesion

## Abstract

Sebaceous hyperplasia is a benign condition characterized by an overgrowth of sebaceous glands, often presenting as small, soft, yellowish papules on areas rich in sebaceous glands, such as the face. While the condition is harmless, it can mimic other dermatological conditions, such as basal cell carcinoma. This case report describes a 71-year-old female with sebaceous hyperplasia presenting as wart-like lesions and whiteheads on the forehead and cheek regions. Histopathological examination confirmed the diagnosis, revealing lobules of sebaceous glands arranged around a central duct in a fibrous stroma.

## Introduction

Sebaceous hyperplasia is a common benign skin lesion resulting from an enlargement of sebaceous glands, typically occurring in older individuals. It manifests clinically as yellowish, umbilicated papules often seen on the forehead, cheeks, and other areas rich in sebaceous glands [1]. The surface of the lesion is smooth with a central area of umbilication due to the dilation of the excretory duct. The condition is most commonly observed in individuals over the age of 50 years, and while benign, it can sometimes be confused with basal cell carcinoma due to its appearance [1,2].

Sebaceous hyperplasia is characterized by an overgrowth of sebaceous lobules around a central duct, and the condition is often associated with aging, chronic sun exposure, and in some cases, hormonal changes [2]. The prevalence of sebaceous hyperplasia was 10% to 16% in patients on long-term immunosuppressive drugs like cyclosporine therapy [1,3]. Another similar common lesion of sebaceous gland origin is acne vulgaris, which is characterized by inflammation of sebaceous glands and hair follicles due to hormonal fluctuation and bacterial infection [4]. The primary concern for patients is cosmetic, though in some cases, multiple lesions may develop, which could raise concerns [5].

A histopathological analysis is critical for a definitive diagnosis and is required to differentiate sebaceous hyperplasia from sebaceous adenoma, molluscum contagiosum, sebaceous nevus, and verruca vulgaris [1,6].

This case report documents a 71-year-old female who presented with lesions resembling warts and whiteheads on the forehead and right cheek, with histopathological confirmation of sebaceous hyperplasia.

## Case presentation

A 71-year-old female presented to the outpatient department of Saveetha Dental College and Hospitals, Chennai, India, with complaints of wart-like lesions and whiteheads on her forehead. These lesions had been slowly increasing in number over the past few years but were asymptomatic. The patient had no significant history of dermatological issues, no family history of skin cancers, and denied any recent sunburns or sun exposure. Past surgical and medical history was non-contributory. Informed consent was obtained from the patient for examination and further investigations.

**Figure 1:**
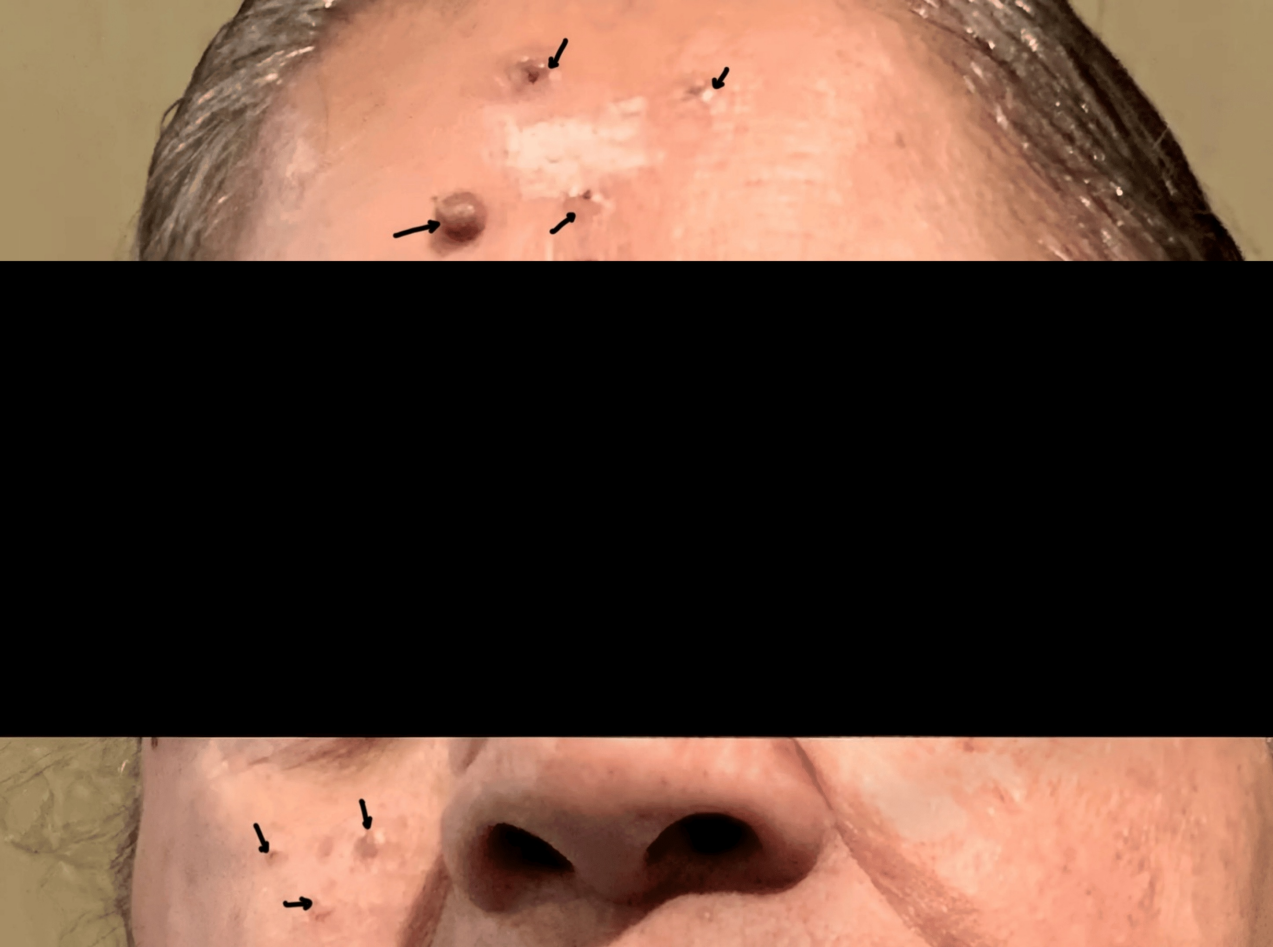
Clinical image Black arrows show blackish-brown papules on the forehead and right malar region

On examination, four small, soft, blackish-brown papules measuring 2-6 mm were observed on the forehead region. The lesions were umbilicated with a slight central depression; the surface was smooth without any ulceration. A few more papular lesions were also noted on the right malar region (Figure 1). No itching or pain was associated with the lesion.

**Figure 2:**
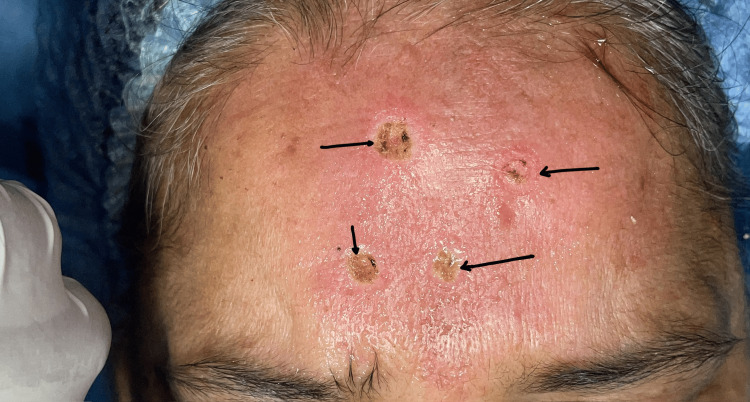
Post-operative image Black arrows showing post-excision sites

Based on the clinical presentation, a differential diagnosis of sebaceous adenoma and verruca vulgaris was considered. An excisional biopsy was performed under local anesthesia (Figure 2) and submitted for histopathological evaluation.

**Figure 3:**
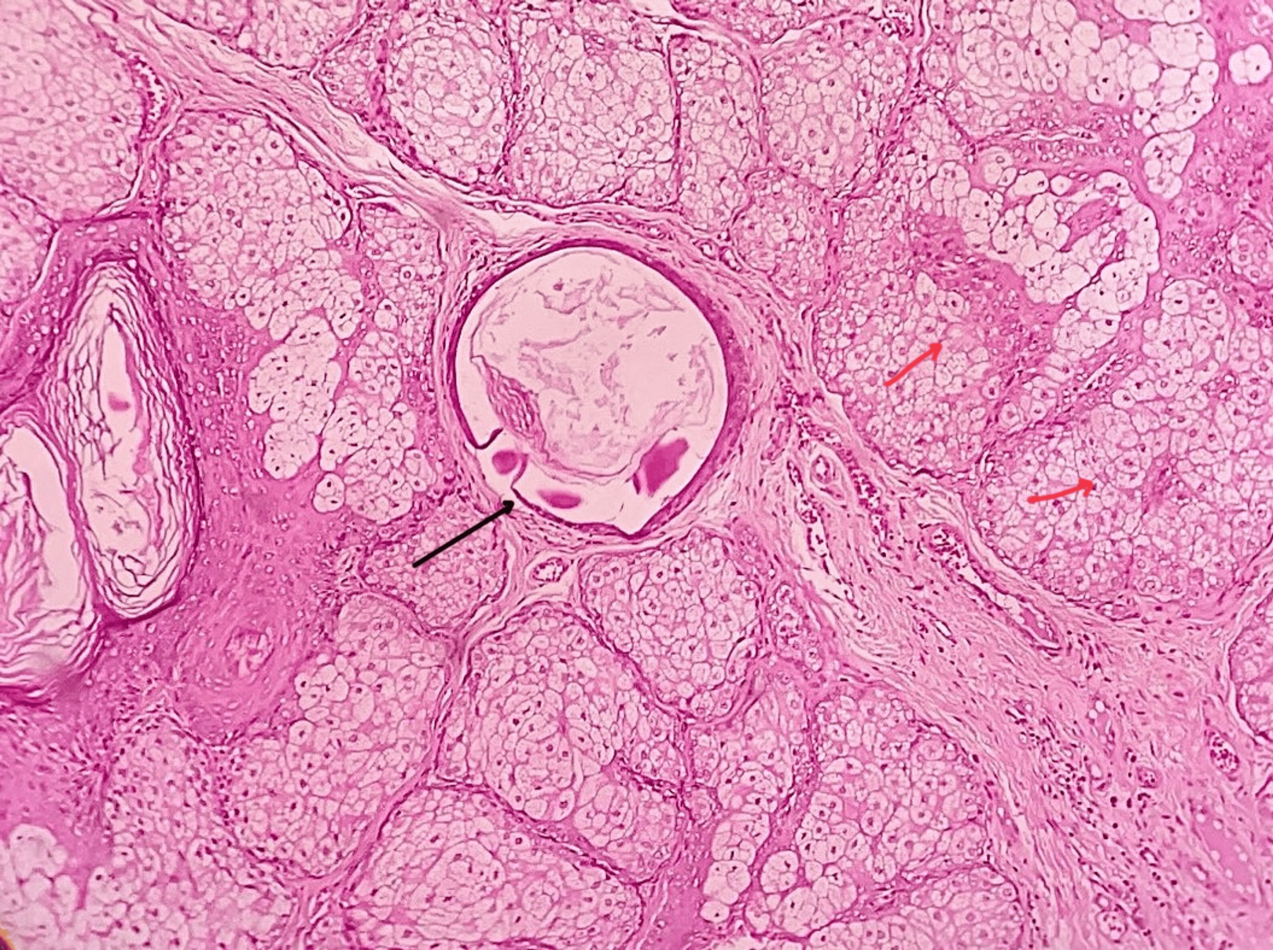
Photomicrograph showing hyperplastic sebaceous glands with ducts (H&E, 20x) Black arrow showing central sebaceous duct. Red arrows showing sebaceous acini around the central sebaceous duct.

Multiple hematoxylin and eosin-stained sections from the biopsy showed a well-circumscribed lesion composed of multiple sebaceous glands arranged in lobules. These lobules contained sebaceous acini with foamy cytoplasm and centrally located basophilic nuclei. The sebaceous lobules appeared grouped around a central sebaceous duct, characteristic of sebaceous hyperplasia. Peripheral basal cells were observed forming a distinct layer around the sebaceous acini. The fibrous connective tissue stroma surrounding the sebaceous lobules showed minimal diffuse chronic inflammatory cell infiltration, predominantly lymphocytes. Blood vessels of varying sizes were present; some were congested with red blood cells. The overlying epidermis was composed of orthokeratinized stratified squamous epithelium, suggestive of normal skin covering the lesion. These histological features were diagnostic of sebaceous hyperplasia (Figure 3).

The patient remains disease-free on 12-month follow-up. She is asymptomatic and satisfied with the treatment outcome.

## Discussion

Sebaceous hyperplasia is a benign condition that primarily affects older adults, typically manifesting in individuals over 50 years of age. This case highlights a 71-year-old female who presented with multiple lesions resembling warts and whiteheads on her forehead and cheeks. The clinical presentation of sebaceous hyperplasia can be subtle, often leading to misdiagnosis of other skin conditions.

Clinically, sebaceous hyperplasia is characterized by small, soft, yellowish papules with a central umbilication. The lesions are typically seen in areas of the face with a high density of sebaceous glands, particularly the forehead and cheeks [3]. Nair and Diwan [7] reported a case of sebaceous hyperplasia over the ear, which clinically mimicked a linear wart over the ear. Our patient presented with papules on the forehead and right cheek region; however, it was not the characteristic yellow color, as reported by previous studies. In this present case, the patient presented with blackish-brown papules that could be attributed to a change in pigmentation and increased melanin production associated with aging skin [8]. It is to be noted that most of the previous studies were carried out with a Caucasian background [2,3,5,6].

Histopathologically, the presence of sebaceous gland lobules with foamy cytoplasm surrounding a central duct is characteristic of sebaceous hyperplasia [7]. Changes in the structure and function of sebaceous glands are linked to levels of circulating androgens. Sebum secretion decreases during childhood, increases at puberty, and reaches its highest level in young adults [8]. While the quantity of sebaceous glands stays consistent throughout life, sebum production typically diminishes in women after menopause. In contrast, men usually experience little change in sebum levels until they reach the eighth decade of life [9]. The pathogenesis of sebaceous hyperplasia is influenced by the hormonal changes that accompany aging [8]. Decreased androgen levels in older individuals result in slower cellular turnover within the sebaceous glands, leading to hyperplasia of the sebaceous glands in the elderly [10].

Additionally, intrinsic factors such as insulin, thyroid-stimulating hormone (TSH), and hydrocortisone are linked to increased proliferation of sebocytes. Extrinsic factors contributing to the proliferation of sebaceous gland hyperplasia include ultraviolet radiation and immunosuppression caused by cyclosporine [9]. Recent molecular studies suggest that the overexpression of age-related genes like Smad7 and parathormone-related proteins is associated with sebaceous hyperplasia, while c-myc overexpression is linked to increased sebum production [11]. In our case, the patient’s age and clinical presentation are consistent with these findings. Chronic sun exposure has also been implicated as a contributing factor; however, our patient did not report excessive exposure.

The main differential diagnosis reported in the medical literature is basal cell carcinoma, especially in elderly patients and in the presence of sebaceous differentiation in histopathology [12,13]. The umbilicated appearance and the occasional presence of telangiectasia in both conditions are significant contributing factors to this confusion. Sebaceous hyperplasia lacks the infiltrative growth pattern and local destructive behavior [12]. Verruca vulgaris was considered due to the presence of wart-like lesions [14]. However, the absence of characteristic koilocytosis and viral inclusions ruled out this diagnosis.

Sebaceous hyperplasia is benign and asymptomatic and does not require treatment. However, various treatment options exist, including cryotherapy, electrosurgery, and laser ablation, though these methods are primarily pursued for aesthetic purposes [1,5,8]. These treatments can be followed up with mesotherapy for enhanced results, as they can aid in skin regeneration with potential anti-aging effects [15], along with platelet-rich plasma (PRP) with microneedling [16]. The prognosis for sebaceous hyperplasia is excellent, with no risk of malignant transformation. However, new lesions may develop over time, particularly in individuals with a predisposition to sebaceous gland overgrowth.

## Conclusions

This case highlights the clinical and histopathological features of sebaceous hyperplasia in a 71-year-old female, emphasizing the importance of distinguishing it from other conditions. Histopathological examination remains the gold standard for diagnosis. Sebaceous hyperplasia is benign and typically requires patient reassurance. Cosmetic treatments are available if desired for the removal of these skin lesions.
